# The influence of 2- chlorodeoxyadenosine in combination with tumour necrosis factor-α or its mutein on murine leukaemias L1210 and P388

**DOI:** 10.1155/S0962935195000330

**Published:** 1995-05

**Authors:** J. Góra-Tybor, K. Warzocha, T. Robak

**Affiliations:** 1 Department of Pharmacology Medical University of Lódź Muszyńskiego 1 Lódź 90-150 Poland; 2 2nd Department of Internal Medicine Medical University of Lódź Pabianicka 62 Lódź 93-513 Poland

## Abstract

We investigated the influence of 2-chlorodeoxyadenosine (2-CdA) in combination with tumour necrosis factor-α (TNFα) or its mutein VI, which differs from the native molecule by N-terminal amino acid composition, on the survival time of mice inoculated with leukaemia L1210 or P388. Groups of mice with leukaemia L1210 and P388 receiving 2-CdA combined with TNFα had shorter survival times than animals treated with these agents separately. In contrast, the administration of 2-CdA in conjunction with mutein VI, prolonged the survival of mice inoculated with these leukaemias as compared with animals receiving these agents separately. The results of the present study emphasize the importance of the biological activity of the TNFα molecule N-terminus.


Mediators of Inflammation 4, 205–208 (1995)

### The influence of 2-chlorodeoxyadenosine in combination with tumour necrosis factor-α or its mutein on murine leukaemias L1210 and P388

J. Góra-Tybor,[1] K. Warzocha[2] and T. Robak[2,CA]

[1]Department of Pharmacology, Medical University of Lódź, Muszyńskiego 1, 90-150 Lódź;

[2]2nd Department of Internal Medicine, Medical University of Lódź, Pabianicka 62, 93-513 Lódź, Poland

[CA]Corresponding Author

**We investigated the influence of 2-chlorodeoxyadenosine (2-CdA) in combination with tumour necrosis factor-α (TNFα) or its mutein VI, which differs from the native molecule by *N*-terminal amino acid composition, on the survival time of mice inoculated with leukaemia L1210 or P388. Groups of mice with leukaemia L1210 and P388 receiving 2-CdA combined with TNFα had shorter survival times than animals treated with these agents separately. In contrast, the administration of 2-CdA in conjunction with mutein VI, prolonged the survival of mice inoculated with these leukaemias as compared with animals receiving these agents separately. The results of the present study emphasize the importance of the biological activity of the TNFα molecule *N*-terminus.**

**Key words:** 2-Chlorodeoxyadenosine, Experimental leukaemias, Interactions of cytokines and antineoplastic drugs, Muteins of tumour necrosis factor-α, Tumour necrosis factor-α.

### Introduction

The combination of cytokines and cytotoxic drugs offers a new approach to increasing the therapeutic effect in the treatment of neoplastic diseases. There are many possible levels of interactions between these two groups of antineoplastic agents: cellular drug uptake, drug target enzymes, drug metabolizing enzymes.

2-Chlorodeoxyadenosine (2-CdA) is a new antineoplastic drug belonging to the purine analogues, which is especially active in lymphoproliferative disorders. Tumour necrosis factor-α (TNFα), a multimodal cytokine, has a well-known antineoplastic activity. Muteins are TNFα derivates, in which the first three to seven amino acids of the native molecule have been replaced. *N*-terminal amino acids play an important role in the binding of TNF to its cell surface receptors and in the biological activity of this cytokine.[1]

The results of our previous experiments showed that both TNFα and 2-CdA administered separately have therapeutic activity against murine leukaemias L1210 and P388.[2,3] Our recent studies have demonstrated that mutein VI has the best therapeutic activity against leukaemia P388.[4]

The present study was designed to compare the influence of 2-CdA combined with TNFα or with its mutein VI, on murine leukaemias L1210 and P388.

### Materials and Methods

*Animals:* For the experiments 140 female mice, DBA/2 strain, weighing 18 – 20 g, 8 – 10 weeks old, purchased from the Institute of Immunology and Experimental Therapy, Polish Academy of Sciences (Wroclaw) were used. They were given standard laboratory food and water *ad libitum.*

*Leukaemia:* L1210 and P388 leukaemia cells were purchased from the Institute of Immunology and Experimental Therapy, Polish Academy of Sciences (Wroclaw) and were maintained by serial passages in the ascitic fluid of DBA/2 mice. Leukaemic cells from the fluid were resuspended in 0.9% sodium chloride so that $10^6$ L1210 or P388 cells were injected i.p. into DBA/2 recipients.

*Therapeutics:* 2-CdA produced according to the method described by Kazimierczuk *et al.*[5] was kindly supplied by the Foundation of Development of Diagnosis and Therapy (Warsaw) as a dry powder. The synthesis and analysis of TNFα and its mutein VI were performed at the Department of Bioorganic Chemistry, Lódź, Poland. TNF had a specific activity of $2 \times 10^7$ U/mg. Mutein VI was constructed using synthetic oligonucleotides to induce changes in the cDNA encoding five *N*-terminal amino acids of native TNF-α.[6] Amino acid sequences were analysed by using automated Edman degradation with an Applied

© 1995 Rapid Communications of Oxford Ltd

**Table 1.** The influence of 2-CdA, TNFα and mutein VI on the survival time of mice with leukaemia L1210

| Therapeutics | NaCl (0.9%) | | | 2-CdA (35 mg/kg)[c] | | |
|---|---|---|---|---|---|---|
| | $x \pm$ S.D.[d] | *MST*[e] | *ILS*[f] | $x \pm$ S.D.[d] | *MST*[e] | *ILS*[f] |
| NaCl (0.9%) | 7.22 ± 0.42 | 7.0 | | 10.35 ± 1.05* | 10.0* | 42* |
| TNF | | | | | | |
| 250 μg/kg/24 h[a] | 9.82 ± 1.22* | 10.0* | 42* | 7.20 ± 0.95 | 7.0 | 0 |
| 400 μg/kg/24 h[b] | 8.20 ± 0.83 | 8.0 | 14 | 6.30 ± 1.25 | 6.5 | −10 |
| Mutein VI | | | | | | |
| 250 μg/kg/24 h[a] | 8.50 ± 0.85 | 8.0 | 14 | 9.50 ± 1.03* | 10.0* | 42* |
| 400 μg/kg/24 h[b] | 9.35 ± 0.90* | 9.0 | 29 | 12.60 ± 0.65* | 12.5* | 78* |

Experiments were performed with groups of six animals. Data represent the average of four identical experiments.
[a]Treatment, once a day, in eight i.p. injections.
[b]Treatment, four i.p. injections on days 2,4, 6, 8 of experiment.
[c]Treatment, once a day, in five i.p. injections.
[d]Mean values and standard deviation.
[e]Median survival time. Six mice were used per group.

$$^{f}ILS = \left( \frac{MST \text{ of the treated group}}{MST \text{ of the control group}} - 1 \right) \times 100(\%)$$

*Statistical significance as compared with the control group. $p < 0.05$.

| | |
|---|---|
| *TNFα* | Met-Val-Arg-Ser-Ser-Ser-Arg-Tyr- |
| *Mutein VI* | Met-Arg-Ile-Arg-Met- |

FIG. 1. The *n*-terminal amino acids sequences of TNFα and its mutein VI.

Biosystems ABI 477A protein sequencer. The cDNA was expressed in *E. coli* and the resulting mutein was purified by ion exchange chromatography. Endotoxin contamination amounted to approximately 1.9 ng endotoxin per mg protein, as estimated using a commercially available assay (Sigma Chemical Co., St Louis, MO, USA). Recombinant cytokines were reconstituted using sterile phosphate-buffered saline (PBS).

Drugs were delivered in a volume of 0.01 ml/g mouse weight. Control mice received equivalent volumes of 0.9% solution of PBS.

*Antileukaemic assay:* Animals received tumour challenges on day 0. All treatments were i.p. and were initiated on the next day. 2-CdA was administered at dose of 35 mg/kg, once a day, for 5 days. Cytokines were administered at dose of 250 μg/kg as daily injections for 8 days or at a dose of 400 μg/kg given 2, 4, 6 and 8 days after the inoculation of leukaemic cells. The control mice received i.p. injections of 0.9% NaCl.

The mice were observed daily for survival for a minimum of 60 days. The median survival time (*MST*) was assessed according to the equation: $MST = (x + y)/2$, where $x$ denotes the earliest day when the number of dead animals was $> N/2$, $y$ denotes the earliest day when the number of dead animals was $> (N/2) + 1$, and $N$ denotes the number of animals in the group. The antileukaemic effect of the drugs (measured as the increase in lifespan, *ILS*) was assessed as the percentage ratio of *MST* of the treated group (T) to that of the control group (C):

$$ILS = [(MST_T/MST_C) - 1] \times 100$$

As each experiment was repeated four times (six mice per group), data are reported for 24 animals per dose group.

*Statistical analysis:* Statistical analysis was performed using Student's *t*-test. Results were considered significant when $p < 0.05$.

### Results

TNFα given once daily, at a dose 250 μg/kg, significantly prolonged the survival time of mice with leukaemia L1210. Nearly the same increase of lifespan in leukemia L1210-bearing mice was seen in the group receiving mutein VI in the sequential treatment schedule. Sequential application of TNFα at a dose of 400 μg/kg and daily application of mutein VI did not change the lifespan of leukaemia L1210-bearing mice as compared with animals of the control group (Table 1).

TNF in both treatment regimes and mutein VI given daily at a dose of 250 μg/kg caused only a slight increase in the lifespan of P388 leukemia-bearing mice. Sequential application of mutein VI

**Table 2.** The influence of 2-CdA, TNFα and mutein VI on the survival time of mice with leukaemia P388

| Therapeutics | NaCl (0.9%) | | | 2-CdA (35 mg/kg)[c] | | |
|---|---|---|---|---|---|---|
| | $x \pm$ S.D.[d] | *MST*[e] | *ILS*[f] | $x \pm$ S.D.[d] | *MST*[e] | *ILS*[f] |
| NaCl (0.9%) | 10.25 ± 0.95 | 10.0 | | 12.50 ± 1.55 | 12.0* | 20 |
| TNF | | | | | | |
| 250 μg/kg/24 h[a] | 10.82 ± 0.75 | 11.0 | 10 | 8.68 ± 0.92 | 9.0 | −10 |
| 400 μg/kg/24 h[b] | 10.70 ± 0.82 | 10.5 | 5 | 9.22 ± 1.18 | 9.0 | −10 |
| Mutein VI | | | | | | |
| 250 μg/kg/24 h[a] | 11.56 ± 1.02 | 11.5 | 15 | 11.73 ± 0.78 | 12.0 | 20 |
| 400 μg/kg/24 h[b] | 13.80 ± 0.86* | 14.0* | 40* | 15.82 ± 0.81* | 16.0* | 60* |

Experiments were performed with groups of six animals. Data represent the average of four identical experiments.
[a]Treatment, once a day, in eight i.p. injections.
[b]Treatment, four i.p. injections on days 2,4, 6, 8 of experiment.
[c]Treatment, once a day, in five i.p. injections.
[d]Mean values and standard deviation.
[e]Median survival time. Six mice were used per group.

$$^{f}ILS = \left(\frac{MST \text{ of the treated group}}{MST \text{ of the control group}} - 1\right) \times 100(\%)$$

*Statistical significance as compared with the control group. $p < 0.05$.

at a dose 400 μg/kg significantly prolonged the survival time of mice with leukemia P388 (Table 2).

The combination of 2-CdA and TNF shortened the survival time of animals in all treated groups. In contrast, mice receiving 2-CdA and mutein VI lived for a significantly longer time than did the animals treated with these agents separately (Tables 1 and 2).

### Discussion

The interactions between cytokines and antineoplastic drugs are very complex. Cytokines can modulate the metabolism of the drug, the expression of adhesion receptors on tumour cells and gene expression.[7] Cytokines also have a great influence on the host's immune system.

TNF is a polypeptide mediator of inflammation and cellular immune response. It also takes part in the induction of antineoplastic activity of the organism. The mechanism of TNF action is still unclear. Numerous different effects of this cytokine may be explained by both structural and functional heterogeneity of TNF receptors and by a diversification of post-receptor signal transduction pathways.[8] Although two cell surface receptors for TNF, p75 and p55, have been identified recently, the amino acid residues necessary for the biological activity of TNF are still not known. Klysik *et al.* constructed derivates of TNF termed 'muteins', in which the first three to seven amino acids of native TNF were replaced using synthetic cDNA expressed in *Escherichia coli*.[6]

The aim of our study was to compare the antineoplastic activity of native TNF + 2-CdA with that of mutein VI + 2-CdA. Our previous results have shown that among muteins III, V and VI, the latter has the greatest activity against murine leukaemias L1210 and P388.[4] Our results have shown that the combination of 2-CdA and TNF shortened the survival time of mice as compared with that of the control group. In contrast, mice receiving 2-CdA and mutein VI lived for a significantly longer time than did the animals treated with these agents separately. These results are in agreement with our previous observations that mice exhibit much better tolerance to high doses of mutein VI as compared with native TNF.[4] From other data[1,9] it is known that *N*-terminal amino acids are critical for both receptor binding and biological activity of TNF. Tchórzewski *et al.* have revealed that mutein VI fails to recognize both TNF receptors. It also fails to increase secretion of cytokines IL-1, IL-6, GM–CSF and IFN-γ, or to activate granulocytes, but it has the highest cytotoxic activity.[1,9] This may suggest an alternative mechanism of action.

TNF may cause tissue damage by augmenting neutrophil function, both by directly activating neutrophils and by affecting their response to other stimuli.[9] The lack of these effects after the addition of mutein VI may explain the lower toxicity when combined with 2-CdA as compared with the combination of native TNF and 2-CdA.

It is well known that TNF acts synergistically with topoisomerase target drugs (adriamycin, amsacrine, mitoxantron, etoposide, teniposide). This effect is probably related to the increase in the activity of topoisomerase I and II, resulting in enhanced DNA strand breaks and cleavage complex.[7] In contrast, TNF does not enhance the antineoplastic effects of bleomycin, hydroxyurea, ara-C, cis-platin, mitomycin, vincristine and vinblastine.[7] Warzocha *et al.* have shown that TNF potentiates the antineoplastic activity of cyclophosphamide and methotrexate.[2] The studies of Winkelhake *et al.* have shown that the combination of TNFα and interleukin-2 does not increase the lifespan of L1210 leukaemia-bearing mice, compared with animals treated with these agents separately.[10] The results of our previous experiments revealed that 2-CdA and interferon-α show an additive inhibitory effect on the growth of clonogenic leukaemic blasts *in vitro*.[11]

The mechanism of action of 2-CdA is still unclear, but it may be related to the activation of apoptosis.[12] Apoptosis (programmed cell death) represents a mechanism of cell removal in embryonic development and in controlling cell populations. Defects of this process may be involved in the production of neoplasms. Apoptosis may be induced by many factors, one of them being TNF.[13,14] TNF-activated protein kinase C appears to be involved in the induction of the c-*jun*, proto-oncogene which seems to be involved in apoptosis.[14–16] Induction of c-*jun* transcription is also observed in the treatment of myeloid leukaemia cells with new purine analogues, which have also been shown to induce apoptosis.[17] These observations may explain the synergistic action of 2-CdA and the TNF derivative, mutein VI.

### References

1. Tchórzewski H, Zeman K, Paleolog E, *et al.* The effects of tumor necrosis factor (TNF) derivatives on TNF receptors. *Cytokine* 1993; **5:** 125–132.
2. Warzocha K, Robak T, Korycka A, *et al.* The influence of tumor necrosis factor α, alone and in combination with cyclophosphamide or methotrexate, on leukemia L1210 and normal hematopoiesis in mice. *Arch Immunol Ther Exp* 1991; **39:** 137–144.
3. Góra-Tybor J, Robak T. Synergistic action of 2-chlorodeoxyadenosine and cyclophosphamide on murine leukemias L1210 and P388. *Acta Haematol Pol* 1993; **24:** 177–182.
4. Warzocha K, Góra-Tybor, J, Robak T, Kwinkowski M. A comparison of the antileukemic effect of recombinant human necrosis factor α and its muteins on leukemia L1210 and leukemia P388 in mice. *Mediators of Inflammation.* 1994; **3:** 411–414.
5. Kazimierczuk Z., Cottam HB, Revankar GR, Robins RK. Synthesis of 2-deoxynucleosides via a novel direct stereospecific sodium salt glycosylation procedure. *J Am Chem Soc* 1984; **106:** 6379–6382.
6. Kłysik J, Konarzewska-Zglińska A, Galazka G, *et al.* Synthesis and expression in *E. coli* of the gene for human tumor necrosis factor. *Arch Immunol Ther Exp* 1991; **39:** 349–356.
7. Kreuser ED, Wadler S, Thiel E. Interactions between cytokines and cytotoxic drugs: putative molecular mechanisms in experimental hematology and oncology. *Sem Oncol* 1992; **19:** (suppl 4): 1–7.
8. Schutze S, Machleidt T, Kronke M. Mechanisms of tumor necrosis factor action. *Sem Oncol* 1992; **19:** 16–24.
9. Tchórzewski H, Zeman K, Kantorski J, *et al.* The effect of tumor necrosis factor α (TNFα) muteins on human neutrophils *in vitro*. *Mediators of Inflammation* 1993; **2:** 41–48.
10. Winkelhake JR, Stampfl S, Zimmerman RJ. Synergistic effect of combination therapy with human recombinant interleukine 2 and tumour necrosis factor in murine tumor model. *Cancer Res* 1987; **47:** 3948–3953.
11. Robak T, Korycka A., Góra-Tybor J. The interaction of 2-chlorodeoxyadenosine (2-CdA) and interferon α (INFα) on normal and myeloid leukemia hematopoiesis *in vitro*. *Leukemia Res* 1994; **18:** 275–281.
12. Robertson LE, Chubb S, Meyn R, *et al.* Induction of apoptotic cell death in chronic lymphocytic leukemia by 2-chloro-2′-deoxyadenosine and 9-β-D-arabinosyl-2-fluoroadenine. *Blood* 1993; **81:** 143–150.
13. Bonnet PA, Robins NK. Modulation of leukocyte genetic expression by novel purine nucleoside analogues. A new approach to antitumor and antiviral agents. *J Med Chem* 1993; **36:** 635–653.
14. Wyllie AH. Apoptosis. *Br J Cancer* 1993; **67:** 205–208.
15. Loetscher H, Steinmetz M, Lesslauer W. Tumor necrosis factor: receptors and inhibitors. *Cancer Cells* 1991; **3:** 221–226.
16. Tartaglia LA, Goeddel DV. Two INF receptors. *Immunology Today* 1992; **13:** 151–153.
17. Brach MA, Herrman F, Kufe DW. Activation of the AP-1 transcription factor by arabinofuranosylcytosine in myeloid leukemia cells. *Blood* 1992; **79:** 728–734.

**Received 14 February 1995; accepted 9 March 1995**
